# Supplementary material for: Perinatal Exposure to Triclosan Results in Abnormal Brain Development and Behavior in Mice
Source: Int J Mol Sci. 2020 Jun 3;21(11):4009. doi: 10.3390/ijms21114009 (PMC7312693; doi:10.3390/ijms21114009)
Supplement: Supplementary file 1 [file ijms-21-04009-s001.pdf]

Supplementary figure-1

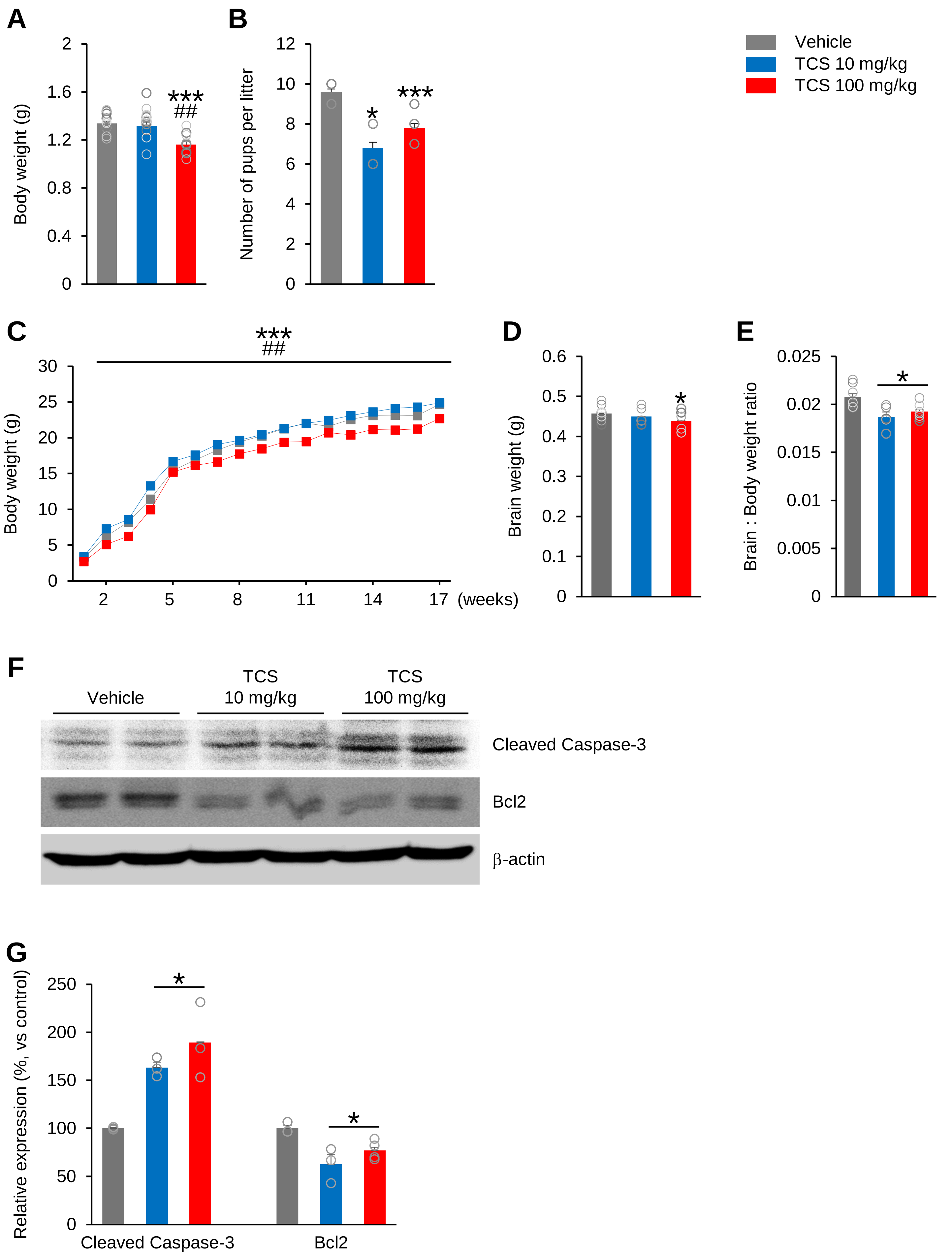

Supplementary figure 1. Impair of TCS on the growth of offspring mice. (**A**) The body weight of offspring mice at PND1. At dose 100 mg/kg, TCS-exposure showed significantly decreased the birth weight compare to vehicle group. There was no change in TCS 10 mg/kg group and vehicle group. (**B**) The numbers of per litter were significantly decreased in the TCS-treated groups compared to the vehicle group. (**C**) The body weight of offspring mice was weekly assessed. TCS 100 mg/kg group delayed the offspring growth and development compared to vehicle group. (**D**) Brain weight of offspring mice at PND 119. There was significantly decreased in the brain weight in TCS 100 mg/kg group compare to vehicle. (**E**) Ratio of brain weight to the whole bodyweight at PND 119. TCS-groups showed significant decreased in the ratio of brain weight to whole body weight compared to vehicle group. Total brain caspase-3 and Bcl2 protein content of adult offspring mice were analyzed through Western blotting, using β-actin as loading control. (**F**) Representation of western blots. (**G**) Quantification of **F**. There were markedly higher in the protein level of cleaved caspase-3 in TCS 100 mg/kg group compared to the TCS 10 and vehicle groups. The expressions of Bcl2 protein were significantly lower in both TCS-treated groups. n= 10 mice (4 male, 6 female) for vehicle, 6 mice (4 male, 2 female) for TCS 10 mg/kg, 9 mice (5 male, 4 female) for TCS 100 mg/kg). Data represent mean ± SEM. Data represent mean ± SEM. Statistical significance was determined by one-way ANOVA with Bonferroni correction. $^{*}p < 0.05$ vs. vehicle, $^{*}p < 0.01$ vs. vehicle, $^{***}p < 0.001$ vs. vehicle, $^{\#\#}p < 0.01$ TCS 10 mg/kg vs. TCS 100 mg/kg. Treatments: corn oil; vehicle, TCS; 10 mg/kg/day, TCS; 100 mg/kg.

Supplementary figure-2

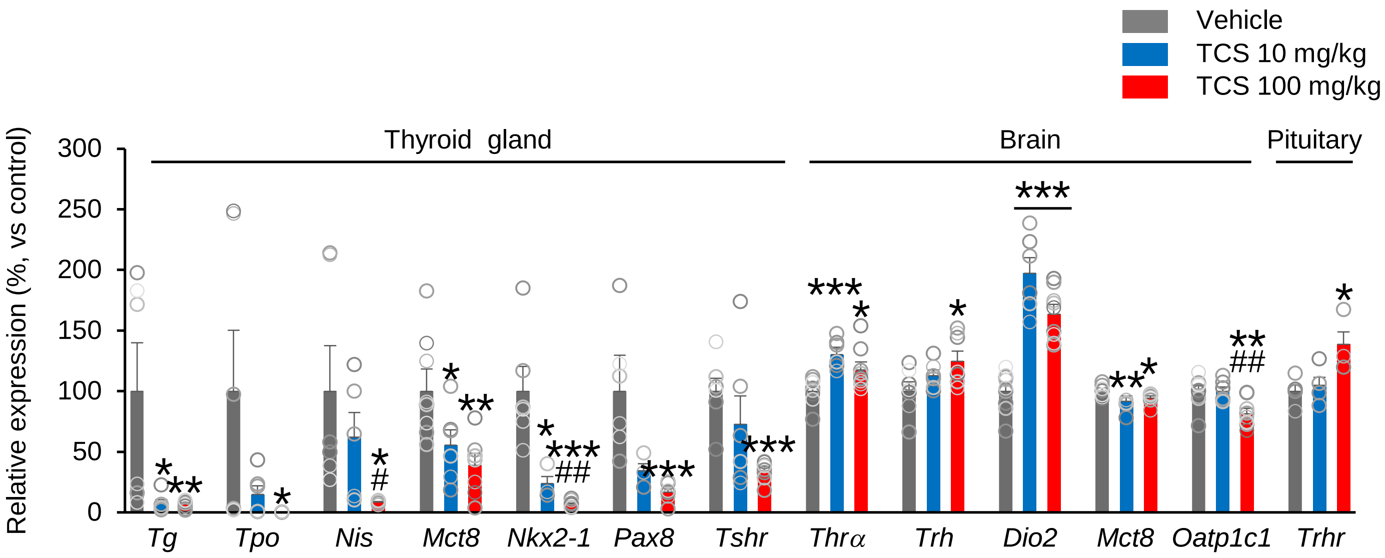

Supplementary figure 2. Impair of TCS on thyroid development and function in mice. Gene expression assessed by realtime quantitative PCR and normalized to *GAPDH* mRNA content in thyroids, brains and pituitary of offspring mice. Thyroid expression of *Tg*, *Tpo*, *Nis*, *Mct8*, *Nkx2-1*, *Pax8* and *Tshr* mRNA levels were reduced in TCS-treated groups compared to vehicle group. Brain *Thr*α, *Trh*, and *Dio2* mRNA levels were higher in TCS-treated groups compared to vehicle group. Whereas, brain *Mct8* and *Oapt1c1* mRNA levels were lower in TCS-treated group compared to vehicle group (n= 10 mice (4 male, 6 female) for vehicle, 6 mice (4 male, 2 female) for TCS 10 mg/kg, 9 mice (5 male, 4 female) for TCS 100 mg/kg). Pituitary *Trhr* mRNA content was increased in TCS 100 mg/kg group compared to vehicle group. n= 5 mice (3 male, 2 female) for vehicle, 4 mice (2 male, 2 female) for TCS 10 mg/kg, 5 mice (3 male, 3 female) for TCS 100 mg/kg. Data represent mean ± SEM. Data represent mean ± SEM. Statistical significance was determined by one-way ANOVA with Bonferroni correction. $^{*}p < 0.05$ vs. vehicle, $^{*}p < 0.01$ vs. vehicle, $^{***}p < 0.001$ vs. vehicle. $^{\#}p < 0.05$ TCS 10 mg/kg vs. TCS 100 mg/kg, $^{\#\#}p < 0.01$ TCS 10 mg/kg vs. TCS 100 mg/kg. Treatments: corn oil; vehicle, TCS; 10 mg/kg/day, TCS; 100 mg/kg.

Supplementary figure-3

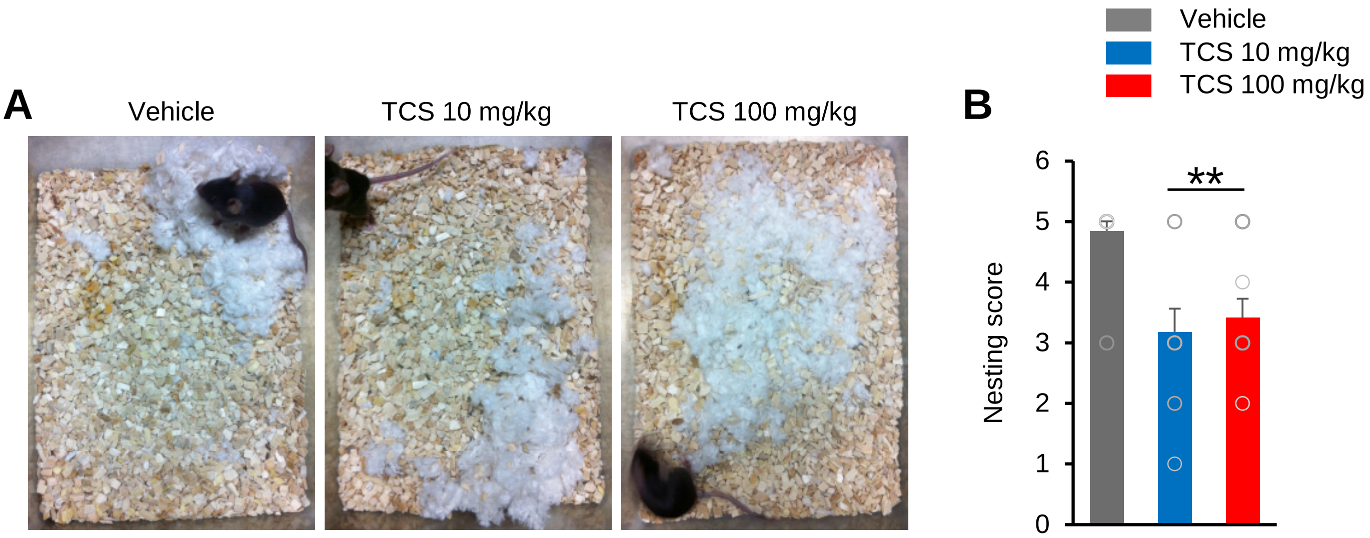

Supplementary figure 3. Impair of TCS on nesting-building behavior in offspring mice. (**A**) Representative images showed the built nest after 12 h after introduction of nesting material. (**B**) Nesting score (mean ± s.e.m.) represents the amount of nesting material used and shape of the nest during the 12-h test period. Score 1 represents an almost untouched nestlet, for score 3 >50% was shredded but not into a specific nest shape, and score 5 represents an almost perfect nest were >90% of the nestlet was used to build the nest as a crater and the walls were higher than the mouse body height. Data represent mean ± SEM. Data represent mean ± SEM. Statistical significance was determined by one-way ANOVA with Bonferroni correction. n= 14 mice (8 male, 6 female) for vehicle, 11 mice (8 male, 3 female) for TCS 10 mg/kg, 13 mice (8 male, 5 female) for TCS 100 mg/kg. $^{**}p < 0.01$ vs. vehicle. Treatments: corn oil; vehicle, TCS; 10 mg/kg/day, TCS; 100 mg/kg.

Supplementary figure-4

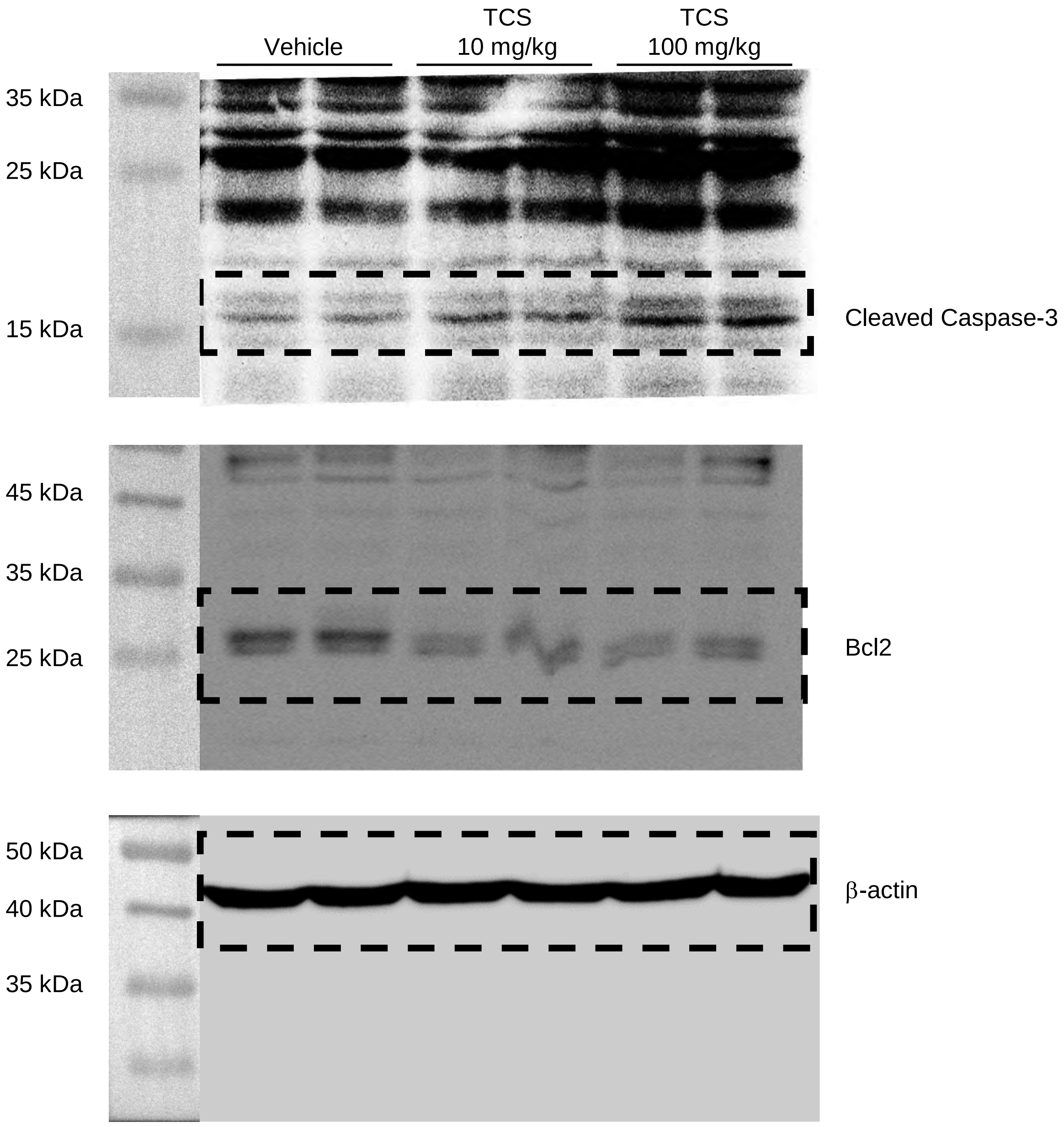

Supplementary figure 4. Western blot images. Western blot images for supplementary figure 1E.

Supplementary Table 1. Primers used for gene expression analysis through Real-Time PCR.

	Forward	Reverse
Tg	ACAGCATTGAAAGGGCGGTA	ATACAAGGTGGTGTCCGCTG
Tpo	GCAGGTGGACACATGCTGATA	CTGGATCCACTTGCAGGTTC
Nis	CCAGTACCTAGAACTGCGCTT	ATGTCCAACCCGGTCACTTG
Mct8	CTTCGGCTGGATAGTGGTGT	GACCCATGCTGCTTGGAACT
Nkx2-1	AACAGAAGTACCTGTCGGCG	CTTAGCCTGGCGCTTCATCT
Pax8	ACTCGATCAGATCCGGCCAT	AATATCACAGGGCCTCACGC
Tshr	CAAAGCTGGATGCTGTTTACCT	GATTTGTGCCTGGTGGAAATCA
Thrα	AAGCTGCTGATGAAGGAGAGAG	CCTGAACAACATGCATTCCGA
Trh	TTGGTGCTGCCTTAGATTCCT	GGCTCCCACTTCTCCCAAAT
Dio2	AACCAAGAGGACCGATGTGC	CGTTCAAAGGCTACCCCGTA
Oatp1c1	TGTAGGGGATTCCAGCTCCT	GTCTGCACACACCCGATGTA
Trhr	CTATGGACATGCTGGCTGCT	CGATGTACCTCTCCACAGTGA
GAPDH	AAGGTCATCCCAGAGCTGAA	AGGAGACAACCTGGTCCTCA

Supplementary Table 2. Summary of behavior statistic data

Test	Duration	Measurement	Number of animals		Values	Statistical test
Morris water maze	1 min	Escape latency (sec)	VE = 18<br>TCS10 = 11<br>TCS100 = 17	Day1	*VE* = 36.79 ± 2.63<br>*TCS10* = 47.39 ± 2.76<br>*TCS100* = 49.51 ± 2.27	One-way ANOVA with Bonferroni correction test
				Day2	*VE* =13.07 ± 1.08<br>*TCS10* = 31.70 ± 2.74<br>*TCS100* = 17.93 ± 1.88	
				Day3	*VE* = 8.64 ±0.73<br>*TCS10* = 16.66 ± 2.31<br>*TCS100* = 15.32 ± 1.90	
				Day4	*VE* = 6.47 ± 0.55<br>*TCS10* = 11.64 ± 1.67<br>*TCS100* = 9.72 ± 1.09	
				Day5	*VE* = 7.10 ± 0.34<br>*TCS10* = 11.59 ± 1.54<br>*TCS100* = 9.54 ± 0.82	
				Day6	*VE* = 6.14 ± 0.45<br>*TCS10* = 8.55 ± 0.87<br>*TCS100* = 8.47 ± 0.86	
				Day7	*VE* = 5.22 ± 0.44<br>*TCS10* = 8.59 ± 0.72<br>*TCS100* = 6.66 ± 0.40	
				Day8	*VE* = 6.47 ± 0.49<br>*TCS10* = 8.66 ±0.83<br>*TCS100* = 9.71 ± 1.39	
				Day9	*VE* = 3.57 ± 0.30<br>*TCS10* = 8.07 ± 0.98<br>*TCS100* = 5.87 ± 0.64	
		Platform crossing			*VE* = 6.06 ± 0.45<br>*TCS10* = 4.45 ± 0.45<br>*TCS100* = 4.70± 0.32	
		Distance move (cm)			*VE* = 730.64 ± 25.90<br>*TCS10* = 681.77 ± 22.86<br>*TCS100* = 713.50 ± 19.54	
		Velocity (cm/s)			*VE* = 12.17 ± 0.42<br>*TCS10* = 11.32 ± 0.37<br>*TCS100* = 11.94 ± 0.31	
Novel object recognition	10 min	Recognition index (%)	VE = 18<br>TCS10 = 9<br>TCS100 = 14		*VE;* Familiar = 45.97 ± 1.71<br>*VE;* Novel = 54.03 ± 1.71	Two-tailed Student's *t* test
					TCS10; Familiar = 48.00 ± 1.62<br>*TCS10;* Novel = 52.00 ± 1.62	
					TCS100; Familiar = 49.39 ± 1.82<br>*TCS100;* Novel = 50.61 ± 1.82	

Social behavior	10 min	Time spent in chamber (sec)	VE = 18<br>TCS10 = 11<br>TCS100 = 17	Sociability	*VE*; Empty = 153.83 ± 5.59<br>*VE*; Stranger I = 274.15 ± 8.67	Two-tailed Student's *t* test
					TCS10; Empty = 186.19 ± 11.16<br>*TCS10*; Stranger I = 237.18 ± 16.23	
					TCS100; Empty = 151.51 ± 7.40<br>*TCS100*; Stranger I = 251.19 ± 13.90	
				Social novelty	*VE*; Stranger I = 149.92 ± 14.48<br>*VE*; Stranger II = 220.07 ± 15.36	
					TCS10; Stranger I = 207.47 ± 11.56<br>*TCS10*; Stranger II = 192.23 ± 19.74	
					TCS100; Stranger I = 183.92 ± 6.94<br>*TCS100*; Stranger II = 212.21 ± 13.12	
Open field	5 min	Time in center (sec)	VE = 18<br>TCS10 = 11<br>TCS100 = 17		*VE* = 14.27 ± 2.17<br>*TCS10* = 13.89 ± 2.95<br>*TCS100* = 7.22 ± 1.17	One-way ANOVA with Bonferroni correction test
		Number of entries into center			*VE* = 11.00 ± 0.82<br>*TCS10* = 12.90 ± 1.63<br>*TCS100* = 5.88 ± 0.64	
Forced swim	5 min	Immobility time (sec)	VE = 18<br>TCS10 = 11<br>TCS100 = 17		*VE* = 188.66 ± 11.08<br>*TCS10* = 189.05 ± 6.95<br>*TCS100* = 191.19 ± 7.92	One-way ANOVA with Bonferroni correction test
Tail suspension	5 min	Immobility time (sec)	VE = 18<br>TCS10 = 11<br>TCS100 = 17		*VE* = 77.56 ± 11.22<br>*TCS10* = 60.21 ± 8.59<br>*TCS100* = 78.78 ± 9.49	One-way ANOVA with Bonferroni correction test
Nesting behavior	12 hour	Nesting score	VE = 14<br>TCS10 = 11<br>TCS100 = 13		*VE* = 4.64 ± 0.27<br>*TCS10* = 3.18 ± 0.38<br>*TCS100* = 3.54 ± 0.33	One-way ANOVA with Bonferroni correction test

VE: Vehicle; TCS10: TCS 10 mg/kg/day; TCS100: TCS 100 mg/kg/day
